# A Model for the Homotypic Interaction between Na^+^,K^+^-ATPase β_1_ Subunits Reveals the Role of Extracellular Residues 221–229 in Its Ig-Like Domain

**DOI:** 10.3390/ijms20184538

**Published:** 2019-09-13

**Authors:** Omar Páez, Marlet Martínez-Archundia, Nicolás Villegas-Sepúlveda, María Luisa Roldan, José Correa-Basurto, Liora Shoshani

**Affiliations:** 1 Department of Physiology Biophysics and Neurosciences, Center for Research and Advanced Studies, CINVESTAV-IPN, Mexico City 07360, Mexico; 2 Laboratorio de Diseño y Desarrollo de Nuevos Fármacos e Innovación Biotécnológica, Sección de Estudios de Posgrado e Investigación, Escuela Superior de Medicina, Instituto Politécnico Nacional, Mexico City 11340, Mexico; 3 Department of Molecular Biomedicine, Center for Research and Advanced Studies, CINVESTAV-IPN, Mexico City 07360, Mexico

**Keywords:** Na^+^, K^+^-ATPase, β-subunit, cell adhesion, protein docking, MD simulations

## Abstract

The Na^+^, K^+^-ATPase transports Na^+^ and K^+^ across the membrane of all animal cells. In addition to its ion transporting function, the Na^+^, K^+^-ATPase acts as a homotypic epithelial cell adhesion molecule via its β_1_ subunit. The extracellular region of the Na^+^, K^+^-ATPase β_1_ subunit includes a single globular immunoglobulin-like domain. We performed Molecular Dynamics simulations of the ectodomain of the β_1_ subunit and a refined protein-protein docking prediction. Our results show that the β_1_ subunit Ig-like domain maintains an independent structure and dimerizes in an antiparallel fashion. Analysis of the putative interface identified segment Lys221-Tyr229. We generated triple mutations on YFP-β_1_ subunit fusion proteins to assess the contribution of these residues. CHO fibroblasts transfected with mutant β_1_ subunits showed a significantly decreased cell-cell adhesion. Association of β_1_ subunits in vitro was also reduced, as determined by pull-down assays. Altogether, we conclude that two Na^+^, K^+^-ATPase molecules recognize each other by a large interface spanning residues 221–229 and 198–207 on their β_1_ subunits.

## 1. Introduction

The Na^+^, K^+^-ATPase or sodium pump (Na^+^-K^+^) is a multimeric protein responsible for the electrochemical gradient of all animal cells. It is composed of α, β and γ subunits. The α-subunits transport the cations and couple them to the hydrolysis of ATP and it is thus considered to be the catalytic subunit of the enzyme [1,2]. Four isoforms of the catalytic subunit are known, α_1_–α_4_ [3]. The β subunit is a glycosylated type II membrane protein. It is necessary for the proper folding, traffic, and insertion of the Na^+^, K^+^-ATPase into the plasma membrane. Also, the β subunit has an influence on the kinetics of K^+^ transport of the α subunit. Three isoforms of β (β_1_–β_3_) of NaK are known [4].

Cell Adhesion Molecules (CAMs) are transmembrane proteins responsible for cell-matrix or cell-cell recognition. Intercellular adhesion via CAMs is a characteristic of epithelial cells [5,6]. The β_1_ subunit of the Na^+^, K^+^-ATPase (NaK β_1_) is a CAM that interacts with another NaK β_1_ on the lateral membrane of epithelial cells. The direct interaction in *trans* of the β_1_ isoform (NaK β_1_-β_1_) in epithelia has been confirmed by co-immunoprecipitation and FRET studies [7,8,9]. This interaction contributes to the polarized expression of the Na^+^, K^+^-ATPase and in turn, to the transporting phenotype of epithelia [10]. The neuronal isoform of the NaK β subunit, β_2_, also acts as an adhesion molecule on glia (AMOG) [11]. However, the interacting partner of β_2_ in *trans* has not been identified but it is probably not a homotypic CAM [12].

The β_1_ subunit of the Na^+^, K^+^-ATPase has a direct role in the formation and stabilization of intercellular junctions in epithelia. It co-localizes with adherens junction proteins since the formation of cellular contacts [13]. In confluent MDCK cells, NaK β_1_-β_1_ interaction increases the stability of adherens junction proteins [8]. The expression of NaK β_1_ subunit is drastically reduced in carcinoma cells. Furthermore, the transfection of NaK β_1_ plus E-cadherin re-establishes the epithelial phenotype of transformed MDCK–MSV cells [14,15,16]. The β_1_ subunit might regulate the γ subunit of NaK (FXYD) and mediate the progression of cancer [17]. The β_1_ subunit is also needed for the correct localization of the Na^+^, K^+^-ATPase and tight junction proteins during blastocyst formation [18]. Even in invertebrates, the β subunit homologue of NaK is essential for the integrity of cellular junctions [19].

The extracellular domain of NaK β_1_ has several N-glycosilation sites [20] and a β-sandwich secondary structure. These are features of CAMs at cell-cell junctions such as cadherins or the immunoglobulin superfamily. Another characteristic of these CAMs is the presence of repeated extracellular domains [21,22,23]. These repeated domains play a key role in the regulation of adhesion in *trans* [24]. In contrast, the extracellular domain of both NaK β_1_ and β_2_ subunits consists of a single globular domain. Several studies have investigated how NaK β_1_-β_1_ interaction occurs at the molecular level. It is known that recognition takes place between amino acid residues and that N-glycans play an important role in its stabilization [8,13]. One study found that segment 198–207 is crucial for the recognition. This was demonstrated through residue substitution by point mutations [25]. Another study identified hotspot residues within the 198–207 region, especially Y199 [26].

Nevertheless, a nine-residue segment might not be sufficient for explaining NaK β_1_-β_1_ interaction. Homophilic dimers of classical CAMs with known crystal structures form larger interfaces. For example, the canonical interface of nectins is formed by more than twenty residues per interacting protein whereas the interface of Type-II cadherins is formed by more than thirty residues per protein [27,28]. In fact, interfaces of around fifty residues are average for weak dimers [29]. In agreement with this idea, mutations in loop 198–207 do not completely abolish NaK β_1_-β_1_ association in vivo [25]. 

Here, we have used bioinformatics tools to identify potential residues involved in the NaK β_1_-β_1_ interaction. First, we performed Molecular Dynamics (MD) simulations and protein-protein docking of the NaK β_1_ extracellular domain. The resulting dimer models consistently included region 221–229 at the interface. Site-directed mutagenesis and adhesion assays suggest that residues along the 221–229 segment are crucial to the recognition between NaK β_1_ subunits. We propose that the Na^+^, K^+^-ATPase associates in *trans* mainly through residues 198–207 and 221–229 in its β_1_ subunit.

## 2. Results

### 2.1. Generation of the Dog Β_1_ Subunit Extracellular Domain Model

**Figure 1:**
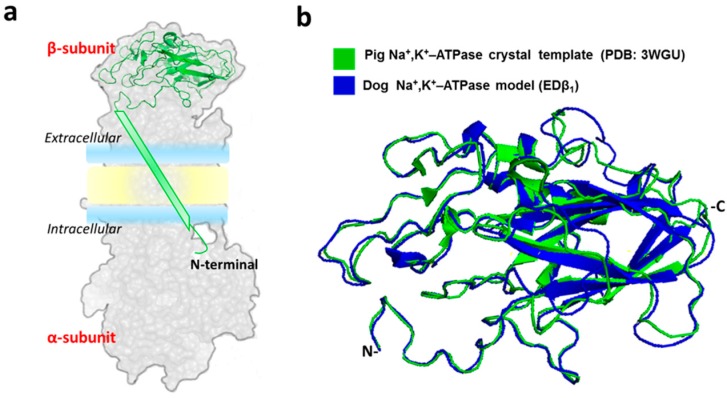
Generation of a high-quality model of canineβ1subunit extracellular domain. (**a**) The silhouette of the complete pig Na^+^, K^+^-ATPase structure (PDB ID = 3WGU). The β1 subunit structure is emphasized in green (cartoon model). (**b**) Structural alignment between the pig (green) and the canine (blue) β1 subunit extracellular domain, named EDβ1. Our model starts from threonine 60 where the single α-helix of TM domain of β1 the ends.

In our laboratory, we have studied the adhesive function of NaK β_1_ subunit in the epithelial MDCK cell line derived from dog kidney [7,8,10,13]. We decided to model a β_1_ subunit with the canine sequence to be able to use our experimental tools for validation of any prediction *in silico*. Several high-quality X-ray structures of the Na^+^, K^+^-ATPase are available at the PDB. We chose the crystal structure of the pig Na^+^, K^+^-ATPase with bound Na^+^ as a template (PDB code 3WGU) because of its high resolution (2.8 Å) and high identity to the canine sequence (92%) [30]. We considered only the extracellular region of NaK β_1_ subunit so we omitted the transmembrane and cytoplasmic domains. The resulting model was designated EDβ_1_ (Figure 1). Validation criteria such as Ramachandran plot showed that the EDβ_1_ model is of very high quality (Supplementary Figure S1). We proceeded to perform MD simulations of the EDβ_1_ model to evaluate its structural stability.

### 2.2. Molecular Dynamics Simulations of the EDβ_1_

**Figure 2:**
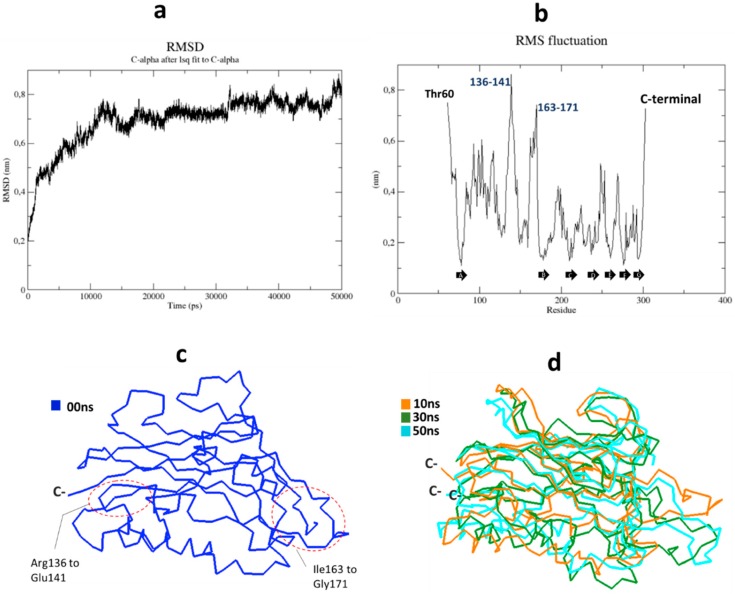
Molecular dynamics simulations of EDβ_1_. (**a**) RMSD (Root Mean Square Deviation) plot of C_α_ computed during 50ns for EDβ_1_. (**b**) RMSF (Root Mean Square Fluctuation) plot of EDβ_1_. Besides C and N terminal ends, segments with the highest fluctuation correspond to residues R136–E141 and I163–G171, respectively. Black arrows indicate the β-sheet secondary structure. (**c**) Ribbon representation of EDβ_1_ before the simulation (native structure, blue). The structure suffers important RMS fluctuation in two loops (red ovals) corresponding to those highlighted in Figure 2b. (**d**) Conformers of EDβ_1_ at the beginning (10 ns conformer, orange), middle (30 ns conformer, green) and end of the simulation (50 ns conformer, cyan).

The stability of the system during MDS was assessed by plotting root mean square deviation (RMSD). Protein flexibility was evaluated by plotting root mean square fluctuation (RMSF). The RMSD plot shows that EDβ_1_ reached equilibrium at about 50 ns of MDS (Figure 2a). The backbone is stabilized from 10 ns to 50 ns (0.2–0.7 nm) suggesting that conformational changes take place from 0 ns to 10 ns as the system equilibrates. The RMSF plot shows that the most stable segments correspond to the β-sheet secondary structure that forms the β-sandwich core of the ectodomain (Figure 2b, black arrows). The most flexible regions of the ectodomain correspond to N and C terminal ends, as expected. Also, loop residues Arg136–Glu141 and Ile163–Gly171 showed high flexibility of around 5.5 to 8.5 Å (Figure 2b). For comparison, we took a sample of five conformers from the MDS trajectory. These structures corresponded to 10 ns, 20 ns, 30 ns, 40 ns and 50 ns of simulation. The structural alignment of the conformers shows that the globular structure of the ectodomain is preserved (Figure 2c,d). These results validated our model and suggested that a ‘soluble’ ectodomain of NaK β_1_ is structurally stable. We then designed a refined protein-protein docking prediction using the different EDβ_1_ conformers. Protein conformers from MD simulations are used to improve docking predictions [31,32].

### 2.3. Protein-Protein Docking Predicts that Na^+^, K^+^-Atpase Β_1_ Subunit Dimer Is Formed by Regions Leu196–Tyr207 and Lys221–Tyr229

**Figure 3:**
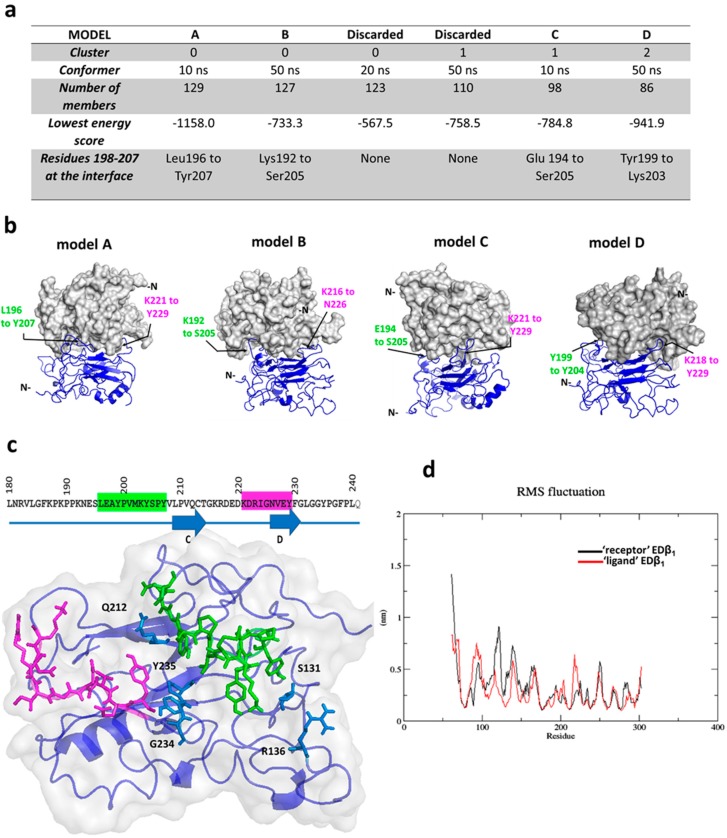
Docking of EDβ_1_ and description of the interface. (**a**) Docking results for the top six largest clusters. (**b**) Models for each of the selected four dimers. One EDβ_1_ is depicted in surface representation (gray) and the docked EDβ_1_ in cartoon representation (blue). Black arrows identify amino acid residues within the same segments (magenta for Lys221–Tyr229 and green for Leu196–Tyr207). (**c**) The interface of model A. The two main segments forming the interface are marked in the β_1_ subunit sequence keeping the same color code as in (**b**). Interface residues highlighted in sticks representation. Light blue residues are those that appear at the interface but are non-continuous in sequence. (**d**) RMSF of model A during 50 ns of Molecular Dynamics simulations.

We chose the ClusPro server because it has been the best predictor in the last rounds of the CAPRI (Critical Assessment of PRediction of Interactions) experiment. This server groups the best 1000 protein complexes in clusters and the size of each cluster is proportional to its probability [33,34]. Prior to docking, we masked residues 61–120 since these are continuous to the TM domain and are facing the plasma membrane in the complete Na^+^, K^+^-ATPase. The six largest clusters are shown in Figure 3a. Computational docking is unable to identify the protein complex closest to the native structure in most cases. Thus, the selection of the best models cannot be based on scoring functions only [34]. In our case, we based the selection on the fact that residues 198–207 must appear at the interface [25]. We analyzed the interface of each of the selected top six complexes to identify residues lying in this segment. We selected four out of the top six models and named these A, B, C and D. Two clusters corresponded to the 10 ns conformer (129 and 98 out of 1000 complexes) and two clusters corresponded to the 50 ns conformer (127 and 86 out of 1000 complexes) (Figure 3a).

The best dimer model, model A, shows that β_1_ subunits interact in an antiparallel fashion. In the other models, β_1_ subunits display a varying orientation. In addition to the 196–207 segment, we identified residues from the region 221–229 at the interface of the four final models. (Figure 3b). This consistency in the docking results suggests that region 221–229 might include potential residues for NaK β_1_-β_1_ interaction. Figure 3c shows the interface of model A in detail. This interface has a buried surface area of 1600 Å^2^. As in the other models, segments Leu196–Tyr207 and Lys221–Tyr229 from both NaK β_1_ subunits form the interface. Residues Ser131, Arg136, Gln212, Tyr235, and Pro236 are also part of the interface but are non-continuous in sequence. We submitted model A to MD simulations to refine and validate the complex. The RMSF plot shows that the C-terminal ends greatly reduced their flexibility in comparison to the monomer (from 7.5 to 4.5–5.0 Å) (Figure 3d). This was expected since β_1_ subunits associate via their C-terminal domains. The same amino acids (136–141 and 163–171) with high deviation in the RMSF of the monomer (see Figure 2b) are highly flexible in the ‘receptor’ β_1_ subunit in the complex (Figure 3d). Supplementary Figure S2 shows the radius of gyration and RMSD plots.

### 2.4. Expression of YFP-β_1_ Mutants in CHO Fibroblasts

**Figure 4:**
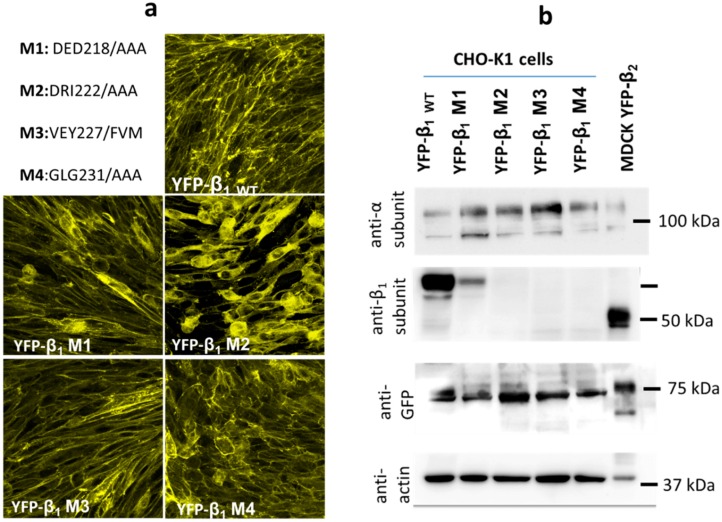
Stable expression of canine YFP-β_1_ subunit mutants in CHO fibroblasts. (**a**) Scheme describing the location of the mutated sites. Three consecutive residues were mutated in each case. Images shown correspond to YFP fluorescence from clones expressing each of the mutants in a stable manner. (**b**) Identification of YFP-β_1_ fusion proteins by western blot: A band of 110 kDa is detected in lysates from cells stably expressing either YFP-β_1_ WT (lane 1) or YFP-β_1_ M1-M4 (lanes 2 to 5) or YFP- β_2_ subunit, with an anti-α subunit antibody (upper panel). In the second panel, the antibody we have regularly used for detection of the canine β_1_ subunit recognizes a band of 75 kDa in cell lysates from YFP-β_1_ WT (lane b) and M1 (lane 2), and a band of 50 kDa in a lysate from MDCK cells expressing the transfected YFP-β_2_ subunit (lane 6), corresponding to endogenous canine β_1_ subunit. That antibody does not recognize the β_1_ mutants M2, M3, and M4 (Lanes 3–5). A 75 kDa band is detected with an anti-GFP antibody (third panel) in all lanes. Anti-actin has been used as an internal control (lower panel).

We decided to use a directed mutagenesis strategy to assess the involvement of the novel region 221–229 in the NaK β_1_-β_1_ interaction. We generated several triple mutations on a fusion protein YFP-β_1_. The YFP protein allows for identification of the transfected protein in vivo. We mutated residues 218–220 (M1), 222–224 (M2) and 231–233 (M4), to alanine. Changing residues 227–229 (M3) for alanine was predicted to disrupt the β-sheet structure in this region. We decided to replace these residues for their equivalent residues in the dog β_2_ subunit isoform (Figure 4a).

Fibroblast cells are an excellent model to study cell-cell adhesion since they do not form cellular junctions. We transfected the wild-type (WT) and the four YFP-β_1_ mutants (M1 to M4) into CHO-K1 fibroblasts. We obtained stable clones expressing each exogenous fusion protein at the plasma membrane (Figure 4a). These results show that none of the NaK β_1_ subunit mutations resulted in synthesis or folding impairments in vivo. WT and mutant (M1 to M4) fusion proteins were also detected by Western blot from cell lysates. For this assay, we used antibodies against the α_1_-subunit, YFP and the β_1_ subunit. Interestingly, the anti-β_1_ antibody detects only the WT and M1 proteins but does not detect mutants M2, M3, and M4. This indicates that the epitope recognized by this monoclonal antibody starts in residue 221 and extends at least to residue 233. However, an anti-YFP antibody detects a 75 kDa band in all cell lines (WT and M1–M4), corresponding to a β_1_ subunit fused with YFP (Figure 4b). We proceeded to perform cell adhesion assays with mutants M1–M4.

### 2.5. Cho Cells Expressing Yfp-β_1_ m2 and m3 Show Decreased Na^+^,K^+^-Atpase β_1_ Subunit- Mediated Intercellular Adhesion In Vivo

**Figure 5:**
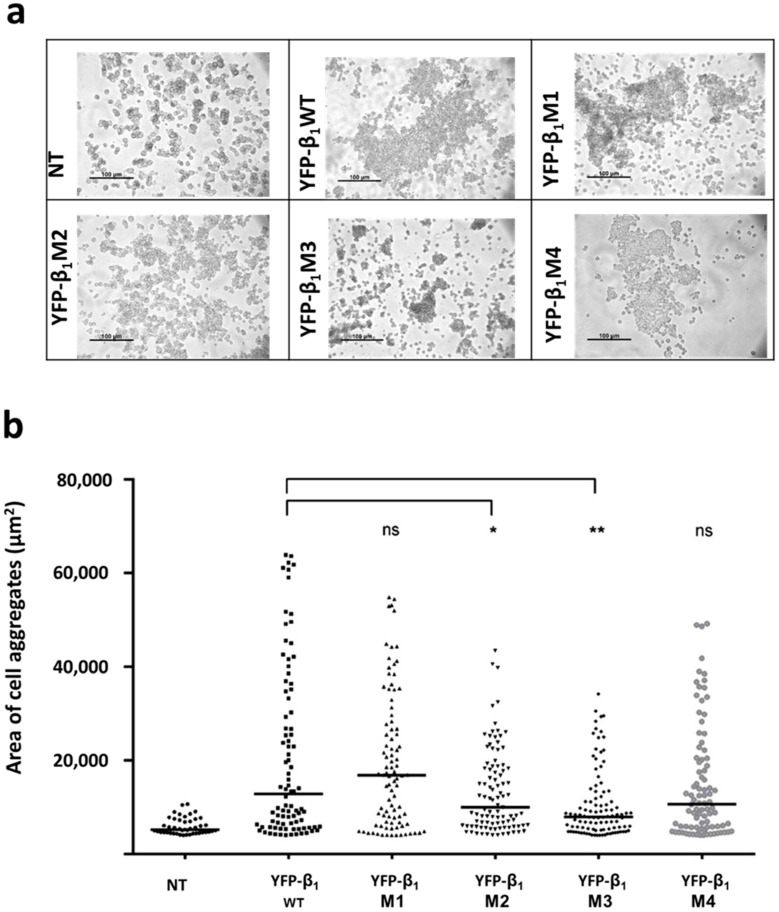
Fibroblasts expressing mutants YFP-β_1_ M2 and M3 show lower adhesiveness. (**a**) Representative snapshots of cells submitted to a Dispase assay. (**b**) Distribution Graph of fragments larger than 5000 μm^2^. Horizontal bars represent median values, (n = 4) *, significant difference *p* < 0.05; **, significant difference *p* < 0.01 and ns = non-significant, non-parametric Mann-Whitney’s test.

As previously shown, transfection of canine NaK β_1_ subunit in CHO fibroblast increases cell adhesion [10,35]. We performed Dispase assays to assess cell-cell adhesion in the stably transfected cell lines. These consist of incubation with the enzyme Dispase to detach the cell monolayer. Resistance to mechanical stress after detachment of the cells can be quantified by measuring the size of the aggregates. After suspension, non-transfected cells (CHO WT) formed many small aggregates. In addition to small aggregates, CHO YFP-β_1_ WT formed cell aggregates of a larger area (Figure 5a). Distribution of cell aggregates is shown in Figure 5b. Aggregates with an area smaller than 5000 µm^2^ showed the same frequency between cell lines, so only those above this value were quantified. Cell lines expressing mutants M1 and M4 showed similar aggregate areas compared to those expressing the non-mutated YFP-β_1_ WT. Cell lines expressing mutants M2 and M3 form aggregates of a smaller area in comparison to cells expressing the non-mutated YFP-β_1_ WT. Nevertheless, none of the four mutant cell lines showed a decrease in adhesion to a comparable level of non-transfected cells. These results suggest that residues mutated in M2 and M3 are involved in the β_1_ subunit-mediated cell adhesion in CHO fibroblasts (Figure 5b).

### 2.6. Cho Cells Expressing Yfp-Β_1_ M2, M3 And M4 Show Decreased Na^+^,K^+^-Atpase Β_1_-Β_1_ Interaction In Vitro

We have previously reported the use of a canine β_1_ subunit tagged with a hexahistidine tail (β_1_His_6_) in its N-terminal end [7]. The β_1_His_6_ protein immobilized in a Ni-NTA matrix interacts in vitro with the soluble ectodomain of the canine Na^+^, K^+^-ATPase β_1_ subunit (sec β_1_). The protein complex is eluted and analyzed for the presence of the two interacting proteins. This ‘pull-down’ experiment is suitable for analyzing the association of β_1_ subunits of different molecular weight.

**Figure 6:**
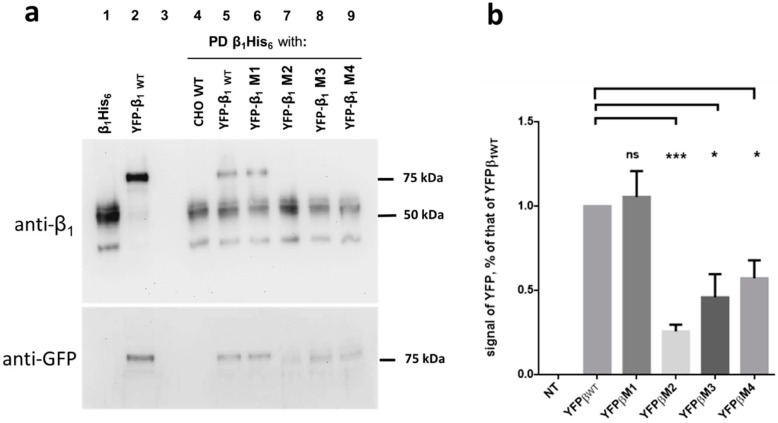
Mutants M2, M3, and M4 show decreased in vitro interaction with an immobilized His_6_-tagged β_1_ subunit. (**a**) Upper panel: an anti-β_1_ subunit antibody detects a ~50 KDa band in a lysate from cells stably expressing the Na^+^,K^+^–ATPase β_1_ subunit with a His_6_ tag (β_1_His_6_, lane 1). The same antibody recognizes a ~75 KDa band in a cell lysate from fibroblasts stably expressing the fusion protein YFP-β_1_ WT. After immobilizing the bait, β_1_His_6_ protein as described in the Methods, total cell lysates from each of the following cell lines were loaded as prey proteins: non-transfected CHO WT cells (negative control) and CHO cells expressing YFP β_1_ WT (positive control) or mutated YFP β_1_ M1–M4. Eluates from each of the six protein-protein interactions were analyzed by western blot and detected with the same anti- β_1_ subunit antibody as in Figure 5 (lanes 4 to 9). Lane 3 was intentionally left empty. A positive pull-down pattern corresponds to the mixture of both the immobilized and prey proteins (lane 5). In a negative pull-down only the immobilized protein was detected (lane 4). (**b**) Densitometric quantification of the pull-down results in A. The signal obtained by anti-GFP antibody in each of the eluted proteins was normalized with the signal of YFP β_1_ WT and depicted as a comparative bar graph. Error bars SD ± (n = 3) *, significant difference *p* < 0.05; ***, significant difference *p* < 0.0005 and ns = non-significant, unpaired Student’s *t*-test with Welch correction.

We wondered whether the fusion protein YFP-β_1_ WT (75 kDa) was capable of interacting with β_1_His_6_ (50 kDa). Representative results are shown in Figure 6a. Incubation of YFP-β1 WT with immobilized β_1_His_6_ resulted in the detection of both proteins in the eluate fraction. This indicates a positive association in vitro (lane 5, upper panel). In contrast, incubation of non-transfected CHO WT cell lysates with immobilized β_1_His_6_ resulted in the detection of only β_1_His_6_ in the eluate fraction (lane 4, upper panel). We incubated lysates from each of the YFP-β_1_ mutant expressing cell lines (M1 to M4) with immobilized β_1_His_6_. As expected, eluates blotted with the antibody against the NaK β_1_ subunits detected a 75 kDa band only for M1 (lane 6, upper panel). As shown in Figure 4b this antibody does not detect M2 to M4. (lanes 7 to 9, upper panel). However, a 75 kDa band was detected for the YFP-β_1_ WT and the four mutant cell lines (M1 to M4) when eluates were blotted with an anti-GFP antibody (lanes 6 to 9, lower panel). We detected different signal intensity for each of the four mutants. The bar graph in Figure 6b shows the densitometry quantification of immunoblots of each YFP-β_1_ mutant in relation to that of YFP-β_1_ WT. YFP-β_1_ M1 showed no difference from non-mutated YFP-β_1_ WT. In contrast, YFP-β_1_ M2 showed a very low signal, a 25% of that of YFP-β_1_ WT. YFP-β_1_ M3 and YFP-β_1_ M4 showed similar levels of approximately 50% decrease in the signal compared to the non-mutated YFP-β_1_ WT.

These results suggest that residues mutated in M2 and M3 are most probably involved in the association between Na^+^, K^+^-ATPase β_1_ subunits. The residues mutated in M4 could also play a role in the recognition between NaK β_1_ subunits, although to a lesser extent.

## 3. Discussion

The β_1_ subunit of the Na^+^, K^+^-ATPase is a homotypic cell adhesion protein in epithelia. The structural basis for NaK β_1_-β_1_ interaction has not been fully described. To our knowledge, this is the first report of MD simulations focused on the Na^+^, K^+^-ATPase β_1_ subunit. Studies of cation selectivity of the Na^+^, K^+^-ATPase have analyzed the structural behavior of the β_1_ subunit transmembrane domain only [36,37]. Our results confirmed that the ectodomain of the β_1_ subunit is a stable structure that remains folded independently of the rest of the Na^+^, K^+^-ATPase. This is consistent with results showing that the ectodomain is resistant to proteolytic cleavage [38]. We also know that a soluble version of the NaK β_1_ ectodomain, named secβ_1,_ maintains its adhesive property [8,26,27]. In our present study, we observed high flexibility in loops Arg136–Glu142 and Ile162–Gly171 during monomer simulation (Figure 2b,d) and dimer simulation (Figure 3d). As already reported, the main residues of the β_1_ subunit responsible for the association with the α subunit are Tyr69 to Tyr83 and Asn182–Ile185 [39]. Thus, this instability is probably due to the fact that our model lacks the structural restrictions imposed by the associated α-subunit. Therefore, the C-terminal half of the ectodomain is more stable than the N-terminal half, which lacks the contact with the α-subunit, and it is in that region that two β_1_ subunits interact to form a *trans* dimer.

Our docking results identified residues Glu194-Tyr207 and residues Lys221-Tyr229, at the interface of the best models of the dimer (Figure 3b,c). The first zone had already been described by Tokhtaeva et al. [25]. Our docking strategy was trustworthy for several reasons: (1) we used a template with a 92% identity. Sequence identity is a reliable indicator of the quality of docking predictions [40]. (2) We used several conformational states as input proteins. This strategy also increases performance [41], and (3) we selected the top models according to experimental evidence.

We assessed the effect of mutations along the segment Lys221–Tyr229 in adhesion assays both in vivo and in vitro. Substitution of residues 218–220 in YFP-β_1_ M1 did not affect NaK β_1_-β_1_ interaction in any experiment. This is consistent with our model since none of the three residues (Glu218, Asp219 and Glu220) is predicted to form the interface. Nevertheless, mutants YFP-β_1_ M2 and YFP-β_1_ M3 (residues 222–224 and 227–229, respectively) showed a significant decrease in adhesion in both assays. These results support the notion that segment 222–229 plays an important role in the recognition, in agreement with our model. Interestingly, the cytoplasmic localization of YFP-β_1_ M2 was higher than all other transfected cell lines (Figure 4a). This might indicate that the membrane stability of the Na^+^, K^+^-ATPase composed of YFP-β_1_ M2 is weakened, but this remains to be investigated. Substitution of residues 231–233 in YFP-β_1_ M4 significantly affected the interaction in vitro but not in vivo. In the second case, only a tendency to decrease was observed (Figure 5 and Figure 6). We propose that mutation of these residues to alanine might have destabilized adjacent residues. In agreement with this idea, adjacent residues Gly234 and Tyr235 both appear at the core of the putative interface (Figure 3c, blue residues). To our surprise, mutation of three consecutive residues did not result in an absolute loss of adhesion neither in vivo nor in vitro. Mutants YFP-β_1_ M2 and YFP-β_1_ M3 both resulted in a decrease in adhesion that was not equivalent to non-adhesive control CHO cells (Figure 5 and Figure 6). However, mutants only targeted half of the putative interface. On this basis, we conclude that residues 222–229 are critical but not sufficient for Na+, K+-ATPase β_1_ subunit association. Supplementary Figure S3 shows polar and hydrophobic interactions between receptor and ligand proteins. Residues Asp222, Arg223, Ile224, Gly225 and Asn226 bind residues along the Val201–Tyr207 sequence, on the homologous β_1_ subunit. This is consistent with Tokhtaeva et al., who identified Leu196, Glu197 Tyr199, and Tyr204 (Tyr 205 in the rat sequence) as hotspot residues [25,26].

In classical CAMs, such as cadherins and nectins, recognition takes place mainly via residues in a β-sheet secondary structure [27,28]. This is to be expected since their extracellular domains are primarily a β-sandwich structure. In contrast, the β_1_ subunit of the Na^+^, K^+^-ATPase has a single globular extracellular domain. According to our model, the adhesive interface of NaK β_1_ is mainly formed by loop residues. It is important to mention that the NaK β_1_-β_1_ interaction is stabilized by the N-glycans on the ectodomain [8,13]. In our model, we did not include any N-glycan moieties; nonetheless, we identified Asn193 as the N-glycosylated residue closest to the interface. It is possible that this particular N-glycan contributes the most to the binding process. Lastly, unlike other CAMs, the β_1_ subunit of the Na^+^, K^+^-ATPase forms an obliged dimer with the catalytic α-subunit. It is expected that the β_1_ subunit suffers conformational changes in response to changes in α-subunit, during the catalytic cycle [42]. There are reports of MD simulations of the complete Na^+^, K^+^-ATPase [43,44]. However, they offer no description of the structural relationship between α and β subunits. Taken together, the results of this study complement our understanding of Na^+^, K^+^-ATPase recognition at the intercellular space. Current work in our laboratory is aimed at characterizing the kinetic properties of dimer formation.

## 4. Materials and Methods

### 4.1. Na^+^, K^+^-Atpase Β_1_-Subunit Modeling

Sequences of Na^+^, K^+^-ATPase β_1_ subunit were retrieved from UniProt database (UniProtKB, ID: P06583) and aligned using BLAST (https://blast.ncbi.nlm.nih.gov/Blast.cgi). The X-ray diffraction structure of Na^+^, K^+^-ATPase was downloaded from the Protein Data Bank. Based on identity and crystal resolution, the pig Na^+^, K^+^-ATPase in the E1 state Na^+^ bound was chosen (PDB code 3WGU) [30]. To build the β_1_ subunit model with the dog sequence the SWISS-MODEL server [45] (Web: https://swissmodel.expasy.org/) was used. To study the protein extracellular surface, the resulting model (polypeptide chain only) was edited. The final PDB file included the extracellular domain of β_1_ subunit from residues 61 to 303. Model quality assessment is described in Figure S1.

### 4.2. Molecular Dynamics Simulations

We performed MD simulations of the model using GROMACS package, by employing the OPLS force field for GROMOS (GROMACS 96) [46]. The box dimension was settled at least 2.0 nm away from the wall of the dodecahedral box with periodic boundary condition, solvated with TIP3 water molecules. The system was neutralized up to 0.15 NaCl. Energy minimization was carried out using the steepest descent method. Berendsen temperature coupling and isotropic pressure coupling were established in order to reach a stable environment (300 K 1 bar). To treat electrostatic and Van der Waals interactions, the particle mesh Ewald (PME) algorithm was applied, using the following values, the cut-off for the short-range VdW (rvdw) was set to 1.0 nm and Coulomb cut-off (r coulomb) at 1.0 nm. All the bond lengths were constrained using the LINCS algorithm [47] and the time step was set to 0.002 ps. The complex was equilibrated for 10 ns, and the MD simulation was run for 50 ns. All structural analyses were carried out by means of the program GROMACS [48]. Snapshots of different MD simulations (10 ns, 20 ns, 30 ns, 40 ns and 50 ns) of the trajectory were retrieved for the analysis of the conformational structure of the protein.

### 4.3. Protein-Protein Docking

The ClusPro Server was used to perform protein-protein docking analysis. ClusPro is based on a three-step algorithm. First, it runs PIPER, a rigid body docking program, based on Fast Fourier Transform (FFT) docking method with pair wise potentials. Then, by using a clustering technique for the detection of near-native conformations [33] and by eliminating some of the non-native clusters, the 1000 best energy conformations are clustered and the 30 largest clusters are retained for refinement. Finally, through Monte Carlo simulations, the stability of each complex is analyzed and finally, structures are refined through SDU (Semi-definite programming based underestimation). Snapshots at different MD simulations (10 ns, 20 ns, 30 ns, 40 ns, and 50 ns) of the trajectory were retrieved and submitted them to molecular docking using the ClusPro server (https://cluspro.bu.edu/login.php) [34]. Output files are grouped into 4 categories: (1) Balanced, (2) Electrostatic-favored, (3) hydrophobic-favored and (4) VdW+Elec. As suggested by the server, we chose complex in the balanced (1) category. All conformers were submitted to a directed docking by masking residues Ile61 to Ser115.

### 4.4. Interface Analysis and Molecular Visualization

We used the server SPPIDER (Solvent Accessibility based Protein-Protein Interface identification and Recognition) for the protein-protein interface analysis. Default settings were at least a 4% change in solvent accessibility (RSA). The dimer was also submitted to InterProSurf (UTMB Health, Galveston, TX, USA), which generates values for solvent accessibility change for each residue in monomeric and dimeric states. Visualization and structural analysis of the models for image design were performed with the package Pymol Molecular Graphics System version 1.7.4.5 (Schrödinger, LLC., New York, NY, USA), academic version.

### 4.5. Site-Directed Mutagenesis

For directed mutagenesis, the plasmid pEYP-C1 (Clontech, Mountain View, CA, USA) with the inserted dog NaK β_1_ sequence (Gene ID: 403966) was used as a template. This plasmid expresses the YFP protein fused to the intracellular N terminal of β_1_ subunit. Plasmid preparation including PCR, restriction assays and selection was as previously reported for the analog pEYP-N1 [7]. The following mutated oligonucleotides were used for triple mutations:

1. 5′-CAGTGCACTGGCAAGCGAGCTGCTGCCAAGGATAGAATTGGGAAC-3′,
2. 5′-CAAGCGAGACGAAGACAAGGCTGCAGCTGGGAACGTGGAGTATTTTG-3′,
3. 5′-CAAGGATAGAATTGGGAACTTCGTGATGTTTGGCCTGGGCGGCTACCC-3′
4. 5′-GGAACGTGGAGTATTTTGCTGCTGCTGGCTACCCGGGCTTTCCTC-3′.

The Quick Site Mutagenesis kit (Stratagene, La Jolla, CA, USA) was used according to instructions. Resulting DNA was transformed into competent *E. coli* cells and DNA sequencing was performed in order to confirm mutations.

### 4.6. Cell Culture, Transfection, and Imaging

CHO-K1 fibroblasts (ATCC CCL-61) were cultured in a mixture of F12\DMEM media (1:1) complemented with a mixture of 100 U/mL penicillin, 100 µg/mL streptomycin and 10% fetal calf serum (GIBCO). For transfection, cells were harvested using trypsin-EDTA and cultured in a serum-free medium on dishes with coverslips at a confluence of 70%; 3 µg of plasmid DNA was transfected according to the lipofectamin 2000 (Invitrogen, Carlsbad, CA, USA) protocol. Cells expressing the transfected protein were selected using G-418 (GIBCO) for the generation of stable clones. For stable clone imaging, cells cultured on coverslips were fixed with cold methanol and blocked with 3% BSA in PBS buffer. After washing, the coverslips were mounted with VECTASHIELD medium (Vector laboratories, Burlingame, CA, USA) and observed with a TCS SP2 confocal microscope (Leica, Hiena, Germany).

### 4.7. Western Blot

CHO cells were washed with PBS and solubilized with radio immune-precipitation assay (RIPA) buffer (RIPA Lysis Buffer System 24948A, Santa Cruz, Dallas, TX, USA). The amount of protein obtained in the resulting lysate was measured (BCA protein assay reagent; Pierce Chemical, Dallas, TX, USA) and then boiled in sample buffer for the subsequent SDS-polyacrylamide gel electrophoresis (PAGE). Proteins were then transferred to a PVDF membrane (Hybond-P; GE Healthcare, Chicago, IL, USA). The proteins of interest were then detected with the specific polyclonal or monoclonal antibodies, followed by species-appropriate peroxidase-conjugated secondary antibodies (Zymed Laboratories, South San Francisco, CA, USA). For detection of the NaK β_1_ subunit, the antibody kindly donated by Dr. Caplan was used as reported in previous works (Padilla et al. 2010). The other primary antibodies used in this study were: anti-GFP rabbit polyclonal antibody sc-8334 (Santa Cruz, Dallas, TX, USA). Anti NaK α_1_ subunit (Abcam, Cambridge, UK) and anti-actin antibody (hybridoma supernatant were donated by Dr. Hernández Hernández, Cinvestav, Mexico City, Mexico). Immobilon Western Chemiluminescent HRP Substrate (Sigma-Aldrich, St. Louis, MO, USA) was used for chemiluminescent detection and the signal was captured with the ChemiDoc™ XRS+ System (BioRad, Hercules, CA, USA).

### 4.8. Dispase Adhesion Assay

Fibroblasts were cultured in 12-well plates and 24 h after reaching confluency, they were washed with PBS three times and incubated with 200 µL dispase I (2.4 U/mL; Sigma-Aldrich) for 40 min. Released monolayer fragments were subjected to mild mechanical stress by pipetting up and down only three times. A 30 µL duplicate sample was taken from each well and mounted for visualization in an Axiovert 200M Fluorescence/Live cell Imaging Microscope (Zeiss, Oberkochen, Germany). A total of 5 random images from each coverslip were taken, and the experiment was performed four times, amounting to forty snapshots per cell line. The number and area of each aggregate were quantified for each snapshot using the program ImageJ. Cell clumps with an area greater than 1000 µm^2^ were considered for data analyses.

### 4.9. Pull-Down Assay

We performed a batch version of the Pull-down assay employed by Padilla-Benavides et al. [7]. Cell lines stably expressing the dog β_1_ subunit with an N-terminal 6His-tag (β_1_His_6_) were cultured and lysed with RIPA buffer complemented with protease inhibitors; 300 µL of Ni-NTA His•Bind^®^ Resin (Sigma-Aldrich,) previously equilibrated with RIPA buffer, was used to immobilize the β_1_His_6_ protein from the lysates. Then, 4 mg of total protein extract was loaded and left to interact for at least 12 h at 4 °C with gentle shaking. After three washes with 500 µL of a 20 mM imidazole solution, 10 mg protein from the different lysates was loaded as prey and left to interact overnight at 4 °C with gentle shaking. After the three washes with 20 mM imidazole, the bound β_1_His_6_ was eluted with 200 µL of a 500 mM imidazole solution. Eluates were loaded on a 10% SDS-PAGE gel and analyzed by immunoblotting. 

## Supplementary Materials

Supplementary Materials can be found at https://www.mdpi.com/1422-0067/20/18/4538/s1. Figure S1: Quality assessment of the dog ED β1 model, Figure S2: MD simulations of model A, Figure S3: The putative interface in detail.
