# Supplementary material for: A Model for the Homotypic Interaction between Na+,K+-ATPase β1 Subunits Reveals the Role of Extracellular Residues 221–229 in Its Ig-Like Domain
Source: Int J Mol Sci. 2019 Sep 13;20(18):4538. doi: 10.3390/ijms20184538 (PMC6770782; doi:10.3390/ijms20184538)
Supplement: Supplementary file 1 [file ijms-20-04538-s001.pdf]

a

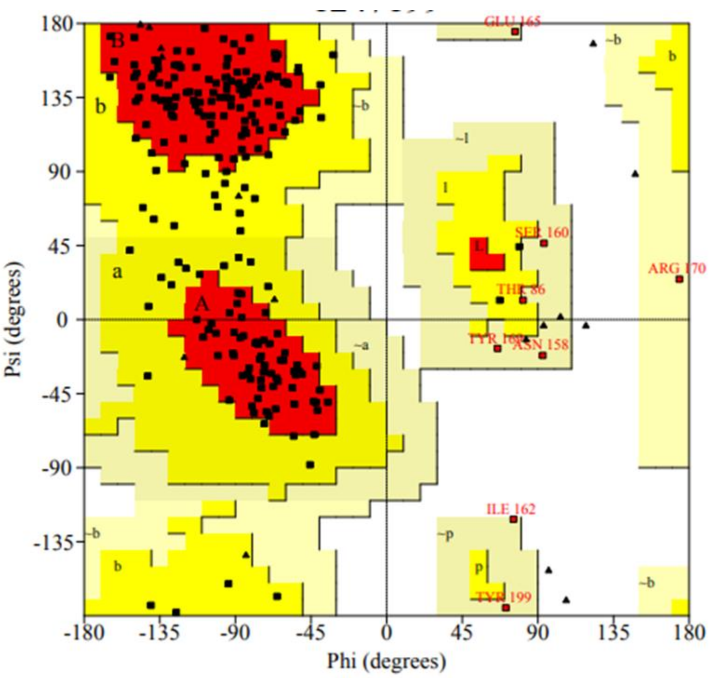

Plot statistics

Residues in most favoured regions [A,B,L]	157	77.3%
Residues in additional allowed regions [a,b,l,p]	38	18.7%
Residues in generously allowed regions [~a,~b,~l,~p]	8	3.9%
Residues in disallowed regions	0	0.0%
	----	------
Number of non-glycine and non-proline residues	203	100.0%
Number of end-residues (excl. Gly and Pro)	2	
Number of glycine residues (shown as triangles)	19	
Number of proline residues	19	

Total number of residues	243	

b

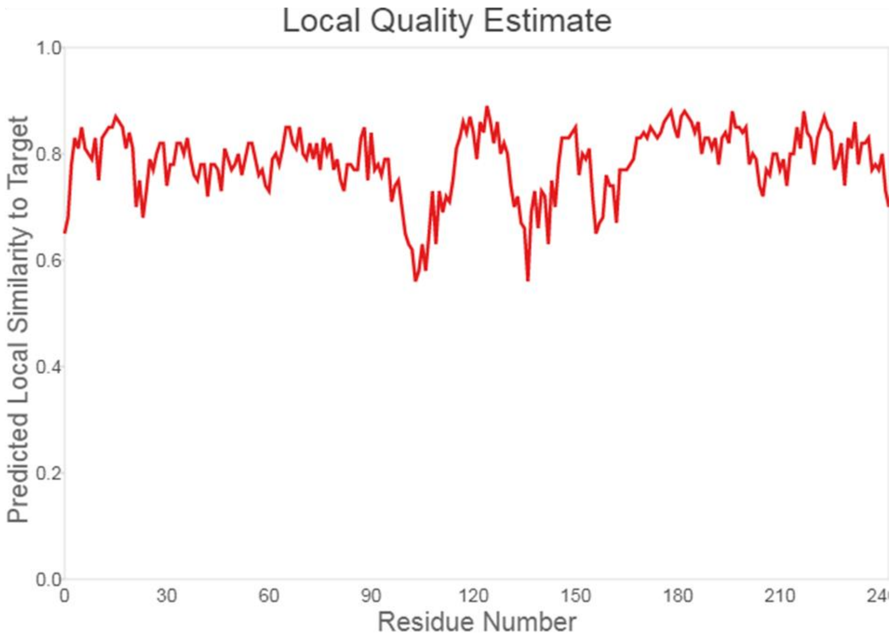

Figure S1. Quality assessment of the dog ED β_1 model.

a) Ramachandran plot for the dog β_1 subunit extracellular domain model ED β_1**.** In ED β_1 77.3% of residues were present in most favourable region, 18.7% in additional allowed region and 3.9% in generously allowed region. No residues were detected in the disallowed region. Plots were generated in the server PROCHECK (https://servicesn.mbi.ucla.edu/PROCHECK/)[49]. **b)** Quality estimate per residue: QMEAN (Qualitative Model Energy ANalysis) is a composite scoring function covering the main aspects of protein stability and describing the agreement of predicted and calculated secondary structure and solvent accessibility[50]. As the reliability of the prediction heavily depends on model size, the provided error estimate is calculated based on models of similar size to the input. ED β_1 had a QMEAN score of 0.78 ± 0.05. Scores of -.4.0 or below are an indication of models of low quality. Evaluation of models is available at the SwissModel workspace (https://swissmodel.expasy.org/)

a **b**

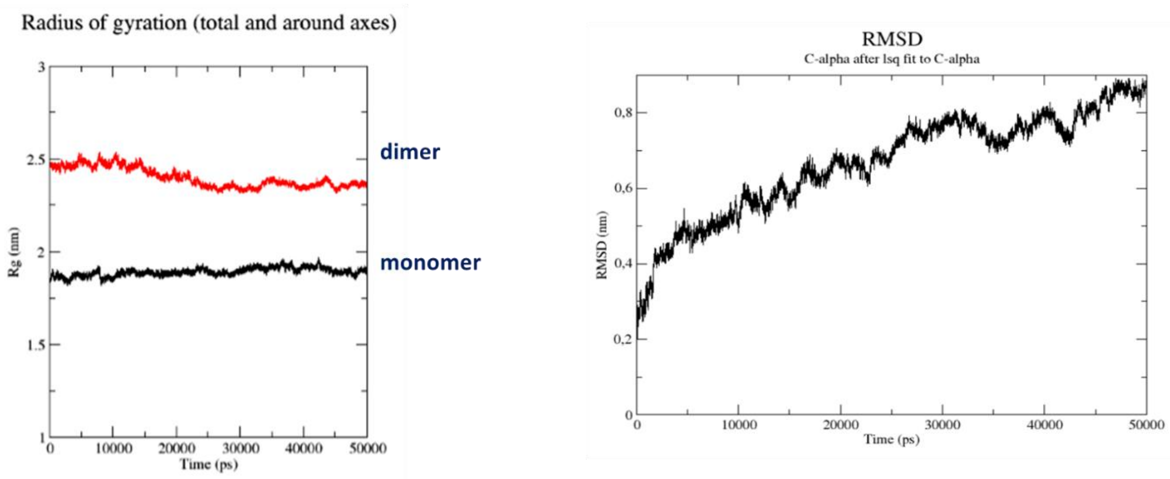

Figure S2. Molecular Dynamics simulations of Model A.

a) Radius of gyration (Rg). Rg is a simple measure of expansion and firmness of the system. The calculated 2D plot for mean Rg of the system was consistent with a stable dimer as compared to the monomer system. **b)** Backbone RMSD scores observed over a period of 50 ns shows that the complex reaches stability by 30 ns of simulation and keeps suffering structural rearrangements by the end of the simulation.

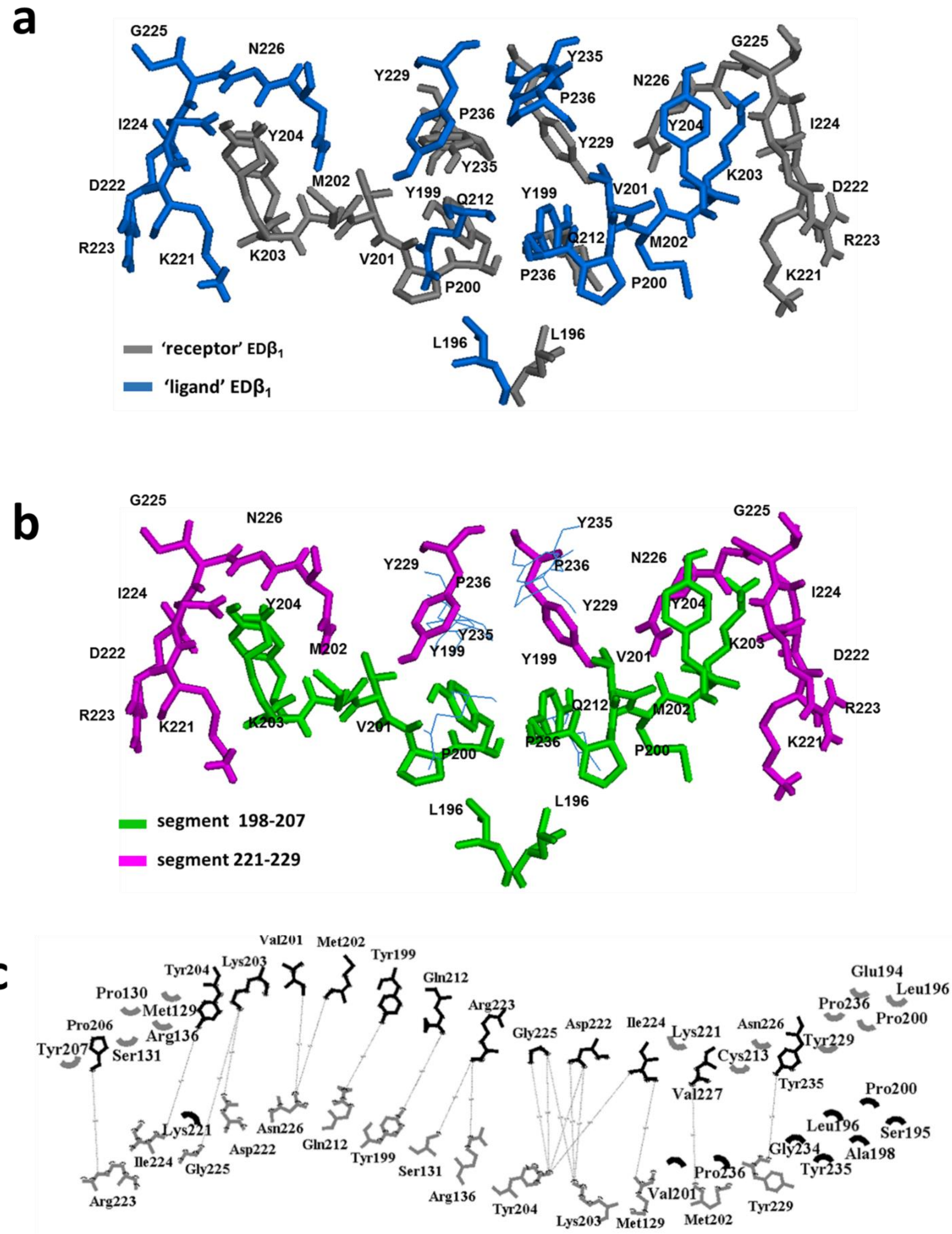
a
G225
N226
Y229
Y235
P236
I224
Y204
K203
D222
M202
Y199
Q212
V201
R223
K221
P200
L196
'receptor' EDβ1
'ligand' EDβ1
b
segment 198-207
segment 221-229
c
Val201
Met202
Tyr199
Gln212
Tyr204
Lys203
Pro130
Met129
Arg136
Pro206
Tyr207
Ser131
Arg223
Gly225
Asp222
Ile224
Lys221
Asn226
Tyr229
Cys213
Val227
Tyr235
Glu194
Leu196
Pro236
Pro200
Ser195
Ala198
Gly234

Figure S3 Detail of the interface in 3D and 2D.

Residues forming the symmetrical interface in model A from the $ED\beta_1$ docking prediction are shown in sticks model distinguishing: **a)** each of the interacting proteins and **b)** the location of segment 221-229 mutated in this study versus 198-207, used to refine the prediction. **c)** Two dimensional model of all residues at the interface generated with the academic version of LigPlot+ program v.4.5.3 LigPlot+[51]. Lateral chain from residues involved in hydrophobic and polar interactions are depicted in sticks model and residues involved in hydrophobic interactions only are depicted with the symbol: . Hydrogen bonds are represented by lines.

References

49. Laskowski, R.A.; MacArthur, M.A.; Moss, D.S.; Thornton, J.M. 20.19. (1993). PROCHECK—A Program to Check the Stereochemical Quality of Protein Structures. *J. Appl. crystallogr.* **1993**, *26*, 283–291, doi:10.1107/S0021889892009944.
50. Benkert, P.; Biasini, M.; Schwede, T. Toward the Estimation of the Absolute Quality of Individual Protein Structure Models. *Bioinformatics* **2011**, *27*, 343–350, doi:10.1093/bioinformatics/btq662.
51. Laskowski, R. A.; Swindells, M B. LigPlot+: Multiple Ligand-Protein Interaction Diagrams for Drug Discovery. *J. Chem. Inf. Model.* **2011**, *51*, 2778–2786, doi:10.1021/ci200227u.
